# Plasmid Construction Using Recombination Activity in the Fission Yeast *Schizosaccharomyces pombe*

**DOI:** 10.1371/journal.pone.0009652

**Published:** 2010-03-11

**Authors:** Ayako Chino, Kenji Watanabe, Hisao Moriya

**Affiliations:** 1 Research Core for Interdisciplinary Sciences, Okayama University, Okayama-City, Okayama, Japan; 2 Precursory Research for Embryonic Science and Technology, Japan Science and Technology Agency, Chiyoda-Ku, Tokyo, Japan; 3 School of Pharmaceutical Sciences, University of Shizuoka, Shizuoka-City, Shizuoka, Japan; University College London, United Kingdom

## Abstract

**Background:**

Construction of plasmids is crucial in modern genetic manipulation. As of now, the common method for constructing plasmids is to digest specific DNA sequences with restriction enzymes and to ligate the resulting DNA fragments with DNA ligase. Another potent method to construct plasmids, known as gap-repair cloning (GRC), is commonly used in the budding yeast *Saccharomyces cerevisiae*. GRC makes use of the homologous recombination activity that occurs within the yeast cells. Due to its flexible design and efficiency, GRC has been frequently used for constructing plasmids with complex structures as well as genome-wide plasmid collections. Although there have been reports indicating GRC feasibility in the fission yeast *Schizosaccharomyces pombe*, this species is not commonly used for GRC as systematic studies of reporting GRC efficiency in *S. pombe* have not been performed till date.

**Methodology/Principal Findings:**

We investigated GRC efficiency in *S. pombe* in this study. We first showed that GRC was feasible in *S. pombe* by constructing a plasmid that contained the *LEU2* auxotrophic marker gene *in vivo* and showed sufficient efficiency with short homology sequences (>25 bp). No preference was shown for the sequence length from the cut site in the vector plasmid. We next showed that plasmids could be constructed in a proper way using 3 DNA fragments with 70% efficiency without any specific selections being made. The GRC efficiency with 3 DNA fragments was dramatically increased >95% in *lig4Δ* mutant cell, where non-homologous end joining is deficient. Following this approach, we successfully constructed plasmid vectors with *leu1+*, *ade6+*, *his5+*, and *lys1+* markers with the low-copy stable plasmid pDblet as a backbone by applying GRC in *S. pombe.*

**Conclusions/Significance:**

We concluded that GRC was sufficiently feasible in *S. pombe* for genome-wide gene functional analysis as well as for regular plasmid construction. Plasmids with different markers constructed in this research are available from NBRP-yeast (http://yeast.lab.nig.ac.jp/).

## Introduction

**Figure 1:**
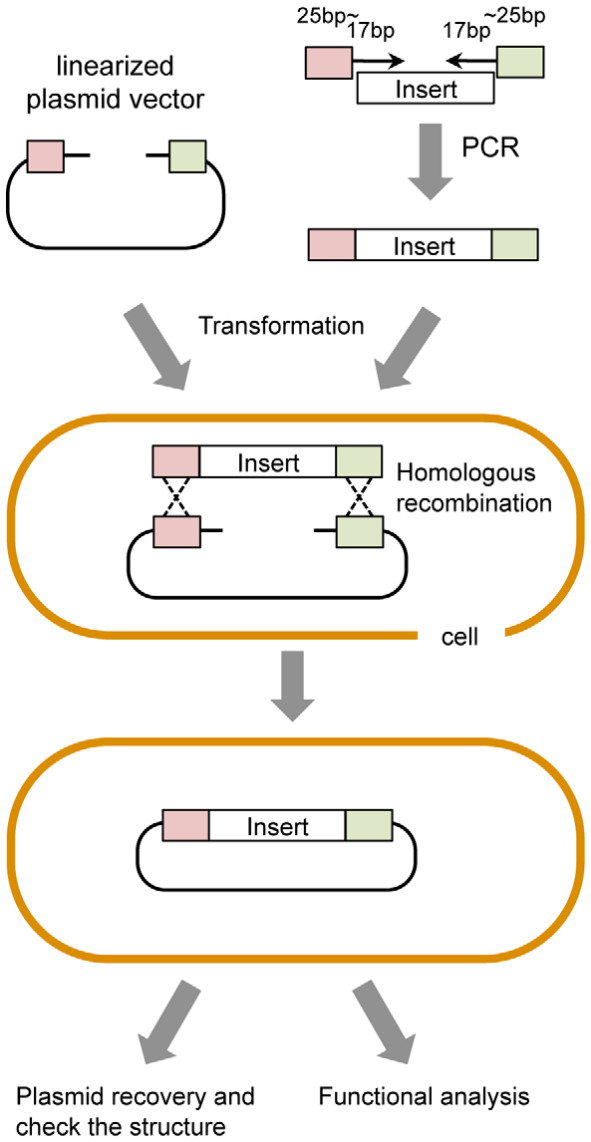
Basic gap-repair cloning procedure. DNA fragment(s) from a linearized plasmid vector are prepared by restriction enzyme digestion or by PCR. DNA fragment(s) from the insert are amplified by PCR, ensuring that the sequence at the end of the fragment is homologous with that of the plasmid. Both DNA fragments are then simultaneously introduced into the cell. Transformants are selected by identifying the plasmid vector marker. The fragments are connected by the homologous recombination activity that occurs within the cell. The constructed plasmid is recovered from the transformed cell into *E. coli* to amplify and check the structure of the plasmid with restriction enzyme digestion and DNA sequencing. Cells with plasmids that have the desired structure are used for functional analysis.

Construction of plasmids is crucial in modern molecular biology. In many cases, plasmids are constructed *in vitro* by digesting (cutting) DNA fragments with restriction enzymes at specific sites (restriction sites) and then ligating (joining) the resulting fragments. The constructed DNA is usually amplified in *E. coli* to analyze its structure. However, a second cloning method using homologous recombination activity (often designated gap-repair cloning or GRC) allows for more design flexibility during construction as restriction sites are not used. The basic GRC procedure is shown in Figure 1. In the budding yeast *Saccharomyces cerevisiae*, efficient GRC is observed due to the high homologous recombination activity of this organism [1], [2], which has been used to construct large-scale systematic plasmid collections [3]–[5]. In these cases, the target genes are directly cloned into *S. cerevisiae* cells to study their function. Thus, GRC is very effective for rapid analyses of gene function.

The fission yeast *Schizosaccharomyces pombe* is also widely recognized as a model eukaryote [6]. Although a previous study has reported GRC feasibility in *S. pombe* [7], it has not been commonly used because there have been no systematic studies reporting its efficiency in this yeast. If GRC in *S. pomb*e is as efficient as it is in *S. cerevisiae*, analyses of gene function using plasmids in *S. pombe* could be performed on a wider scale. Therefore, we studied GRC efficiency in *S. pombe* and first demonstrated amplification of DNA fragments with short homology sequences (>25 bp) by PCR followed by successfully cloning into a *S. pombe* plasmid. We then demonstrated that plasmid construction using 3 DNA fragments could be achieved in 70% efficiency without specific selection for recombination. Using *lig4Δ* mutant cell as a host strain, where the non-homologous end joining (NHEJ) activity is deficient, the efficiency was increased >95%. Thereafter, we constructed plasmids in *S. pombe* with different auxotrophic markers using GRC.

## Results and Discussion

### Cloning the *LEU2* Marker Gene Using GRC

**Figure 2:**
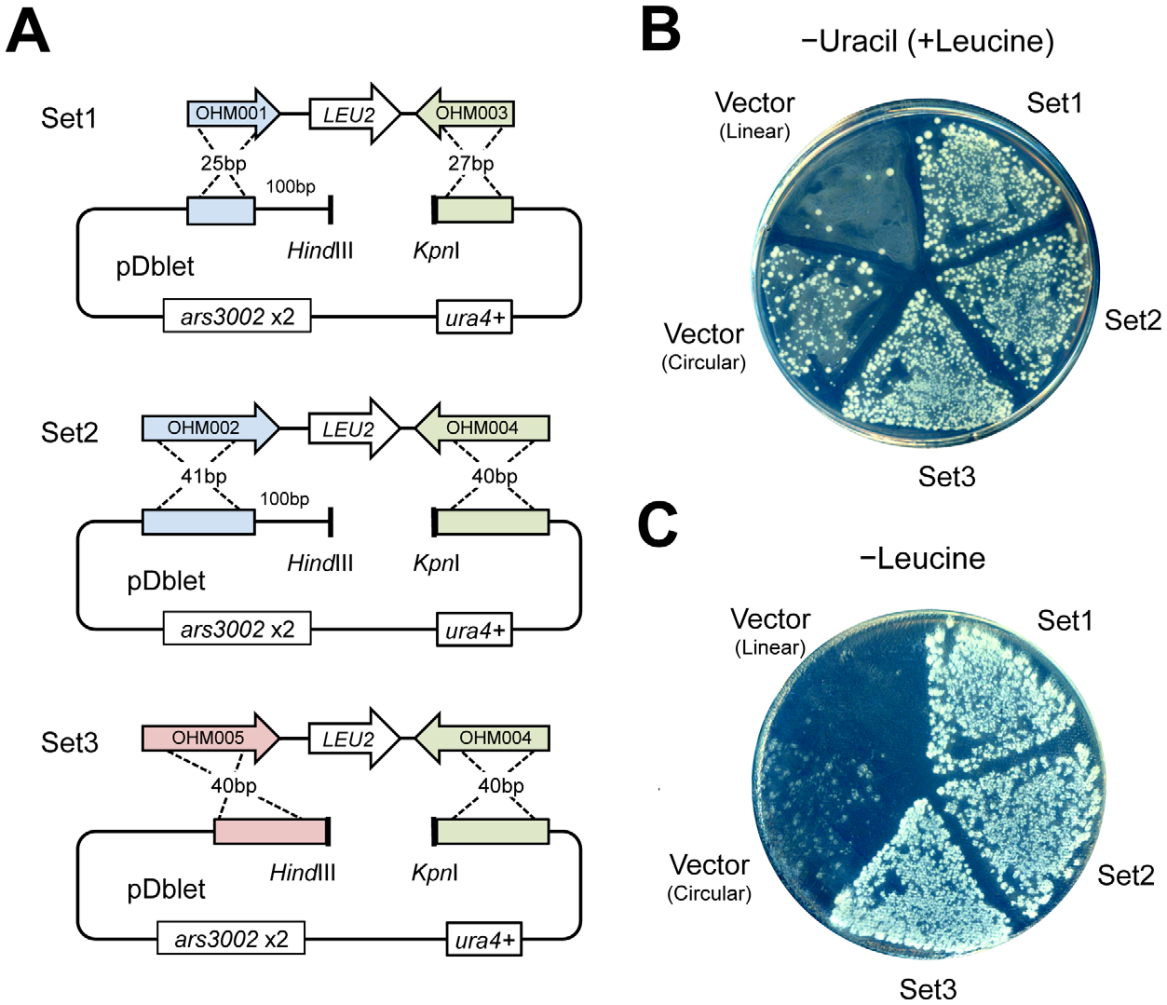
Cloning the *LEU2* marker gene using GRC in *S. pombe*. (**A**) Blueprint of the plasmid construction. DNA fragments containing *LEU2* genes were amplified by PCR using the indicated primer sets with pRS315 as a template. Homologous regions between the vector plasmid (pDblet) and the insert fragments are shown in the same colors. (**B**) Result of transformation with the linearized (*Hin*dIII-*Kpn*I digested) vector, circular vector, and linearized vector with inserts as shown in A. Transformants of Sp286h+ were selected on –uracil plates (EMM with leucine and adenine). (**C**) A replica of the transformants on the plate in B on a –leucine plate (EMM with adenine).

We first evaluated the number of GRC events that could be observed in *S. pombe* and then constructed a plasmid in it using GRC. We used the plasmid pDblet as a cloning vector because it is stable as a monomer and transforms *S. pombe* with high efficiency. It also shows high mitotic stability [8]. These features are advantageous when the constructed plasmid is recovered from the *S. pombe* cells to verify its structure. As inserts, we used DNA fragments containing *S. cerevisiae LEU2* that complemented *S. pombe leu1* so that transformants harboring the plasmids constructed by successful GRC (i.e., pDblet with the *LEU2* gene) could be easily distinguished by replica plating. We also tested the effect of (1) the length of the homologous region (∼25 bp or ∼40 bp) and (2) the distance of the homologous region from the fragment end (Figure 2A).

The fission yeast strain Sp286h+ was transformed with a circular vector, linearized (*Kpn*I-*Hin*dIII digested) vector, and with 3 combinations of linearized plasmids with inserts as shown in Figure 2A. Transformants were first selected by the auxotrophic pDblet (uracil) marker in order to estimate transformation efficiency. As shown in Figure 2B, the linearized plasmids resulted in a lower number of transformants than the circular or the linearized plasmid with inserts. We then tested whether these transformants were leucine prototrophs by replica-plating the transformants onto –leucine plates as this would provide an indirect evidence of successful GRC. Almost all of the transformants from the 3 groups, where the linearized plasmids with inserts were combined, showed leucine synthesizing ability (Figure 2C).

These results suggest that GRC can be successfully performed in *S. pombe*. Because the GRC efficiencies were indistinguishable between the 3 groups where the linearized plasmids with inserts were combined, it was clear that a 25-bp homologous region sufficed for GRC and that the recombination site did not have to be at the end of the fragment. We also constructed linearized vectors by digestion with *Xho*I to check the effect of the restriction sites used. In this case, the number of uracil and leucine-prototroph transformants observed was similar to that observed with the *Hin*dIII-*Kpn*I double-digested vector (data not shown), suggesting that no preferences in restriction site (for single or double restriction enzyme digestions) were observed for GRC in *S. pombe*.

We next examined the structure of the plasmids within transformants grown on –leucine plates by randomly recovering plasmids and digesting them with restriction enzymes and found that >70% of plasmids recovered from the transformants had a desired structure (Table 1). Plasmids without the desired structure appeared to have the *LEU2* gene in the pDblet cloning site but had some mutations in the regions (data not shown). These results indicate that GRC is a useful cloning method in *S. pombe*.

**Table 1:** Structural verification of plasmids found in the transformants after pDblet + *LEU2* construction (Figure 2C).

Insert	No. of positive clones/tested	Positive clone (%)
Set 1	8/11	73
Set 2	9/11	82
Set 3	8/11	73

### Construction of Plasmids Containing *nmt1* Promoter: *EGFP* Using GRC

**Figure 3:**
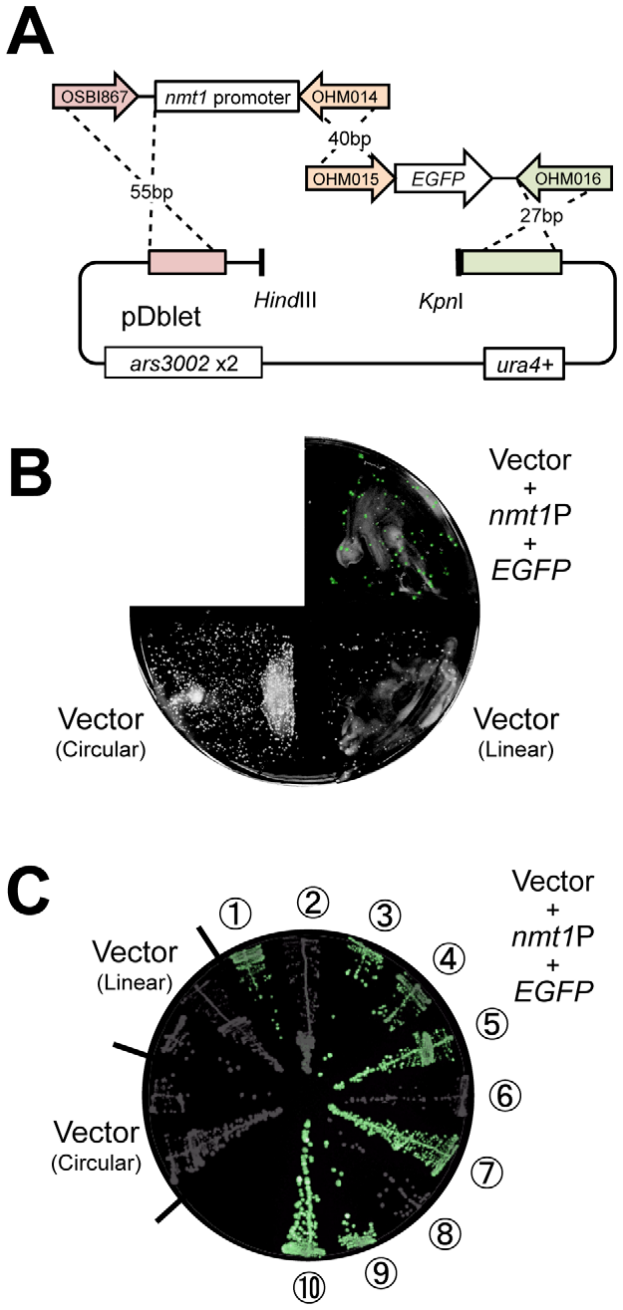
Construction of a plasmid containing *nmt1* promoter: *EGFP* using GRC in *S. pombe.* (**A**) Blueprint of the plasmid construction. DNA fragments containing *nmt1* promoter and *EGFP* were amplified by PCR using the indicated primer sets with pHM004_nmt1-1 and pKT128 as templates, respectively. Homologous regions between the vector plasmid (pDblet) and the insert fragments are shown in the same colors. (**B**) Result of transformation. Transformants of Sp286h+ were selected on –uracil plates (EMM with leucine and adenine). The fluorescent GFP image was superimposed on a bright-field image. (**C**) Independent colonies from plate B were streaked on –uracil plates. The resulting image is shown in B.

We then investigated whether GRC seen in *S. pombe* could be applied in more advanced plasmid constructions. The blueprint of the plasmid construction is shown in Figure 3A. In this process, 3 DNA fragments containing the promoter of the *nmt1* gene, *EGFP* gene, and linearized pDblet are connected. *EGFP* is expressed under the control of the *nmt1* promoter when the plasmid is constructed correctly. As shown in Figure 3B, most of the transformants with the linearized plasmids with inserts showed GFP fluorescence; 7 out of 10 randomly selected transformants showed GFP fluorescence (Figure 3C). One in 10 transformants (Figure 3C 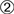 ) showed weak GFP fluorescence.

All plasmids recovered from the 7 transformants showing GFP fluorescence were verified to have the desired structure (Table 2). We could not recover any plasmids from 2 of the 3 transformants showing no or weak fluorescence (Table 2) indicating that the DNA fragments were integrated into the genome in these transformants. The plasmid recovered from the last transformant that did not fluoresce had a truncated insert. To check if the *nmt1* promoter and *EGFP* were joined correctly, we sequenced the connecting region; 2 out of 7 plasmids with the desired inserts had nucleotide substitutions within the connecting region (the primer region for PCR) (Table 2). Currently we are not sure about the reason of these nucleotide substitutions.

**Table 2:** Analysis of independent transformants after pDblet + P*nmt1*-*EGFP* construction (Figure 3C).

Clone No.*1	Fluorescence on plate	Plasmid Recovery*2	Presence of desired insert*3	Presence of mutation*4
1	strong	+	+	−
2	weak	−		
3	strong	+	+	−
4	strong	+	+	+
5	strong	+	+	+
6	no	+	−*5	
7	strong	+	+	−
8	no	−		
9	strong	+	+	−
10	strong	+	+	−

*1. The number corresponds to the number of isolates cloned in Figure 3C.

*2. +: Plasmid is recovered from *S. pombe* in *E. coli*; −: Plasmid could not be recovered from *S. pombe* in *E. coli*.

*3. Presence of desired insert is checked by restriction digestion.

*4. The region between P*nmt1* and *EGFP* is sequenced using OHM025.

*5. The insert is truncated.

### The Efficiency of GRC Is Dramatically Increased in a Mutant Cell without NHEJ Activity

**Figure 4:**
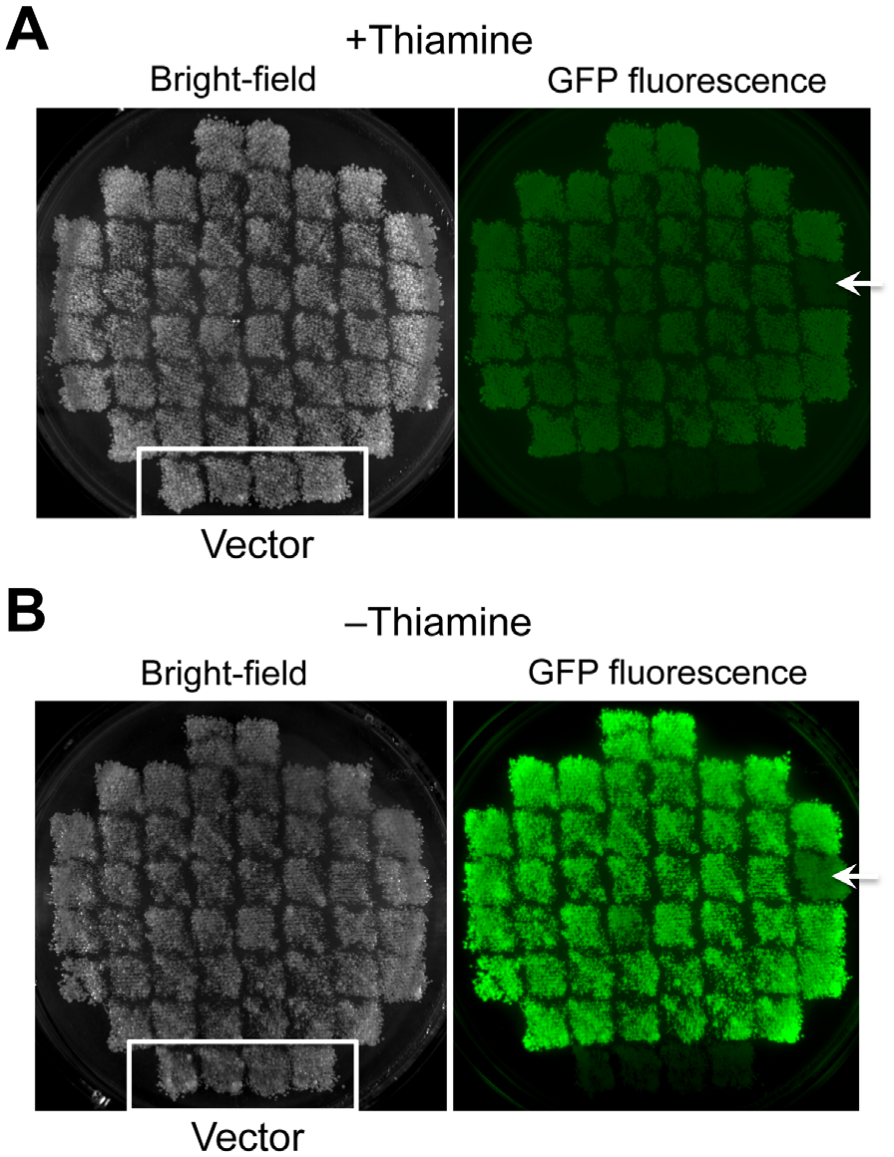
Construction of a plasmid containing *nmt1* promoter: *EGFP* using GRC in the *lig4Δ* mutant *S. pombe*. GRC was performed as in Figure 3 except FY4102 was used as a host strain. Transformants were randomly selected and streaked on –uracil +thiamine plate, then the transformants were replica-plated on +thiamine (**A**) and –thiamine (**B**) plates. Negative controls (transformants with the vector) were also streaked. Arrowheads indicate transformant with weak GFP fluorescence, in which GFP was integrated into genome.

It is reported that the efficiency of homologous recombination event can be increased in a yeast strain without NHEJ activity [9]. We thus tested the GRC efficiency in a *lig4Δ* mutant strain where non-homologous end joining activity is deficient [10]. We performed the same experiment as described in Figure 3 (i.e. DNA fragments containing the promoter of the *nmt1* gene, *EGFP* gene, and linearized pDblet are connected). As shown in Figure 4, 96% (45 out of 46) transformants showed significant GFP fluorescence that was regulated by thiamine, indicating the GRC efficiency was dramatically increased in this strain. In the rest one transformant (arrowhead in Figure 4), GFP was probably integrated into genome because any plasmid could be recovered from the transformant. We recovered the plasmids from the transformants and checked the structure with restriction digestion and sequencing. All recovered plasmid had correct structure, but 3 out of 10 plasmids had nucleotide substitutions within the connecting region (the primer region for PCR). Currently we are not sure about the reason of these nucleotide substitutions.

These results indicate that GRC could be effectively performed in *S. pombe* (with >70% efficiency in wild type, and with >95% in a NHEJ mutant) with 3 DNA fragments without selection for recombination, although constructed plasmids sometimes had nucleotide substitutions within the connecting regions. We thus concluded that GRC is suitable for use as a cloning method in *S. pombe* for genome-wide gene functional analysis as well as for regular plasmid construction.

### Constructions of Plasmids with Different Auxotrophic Markers Using GRC

**Figure 5:**
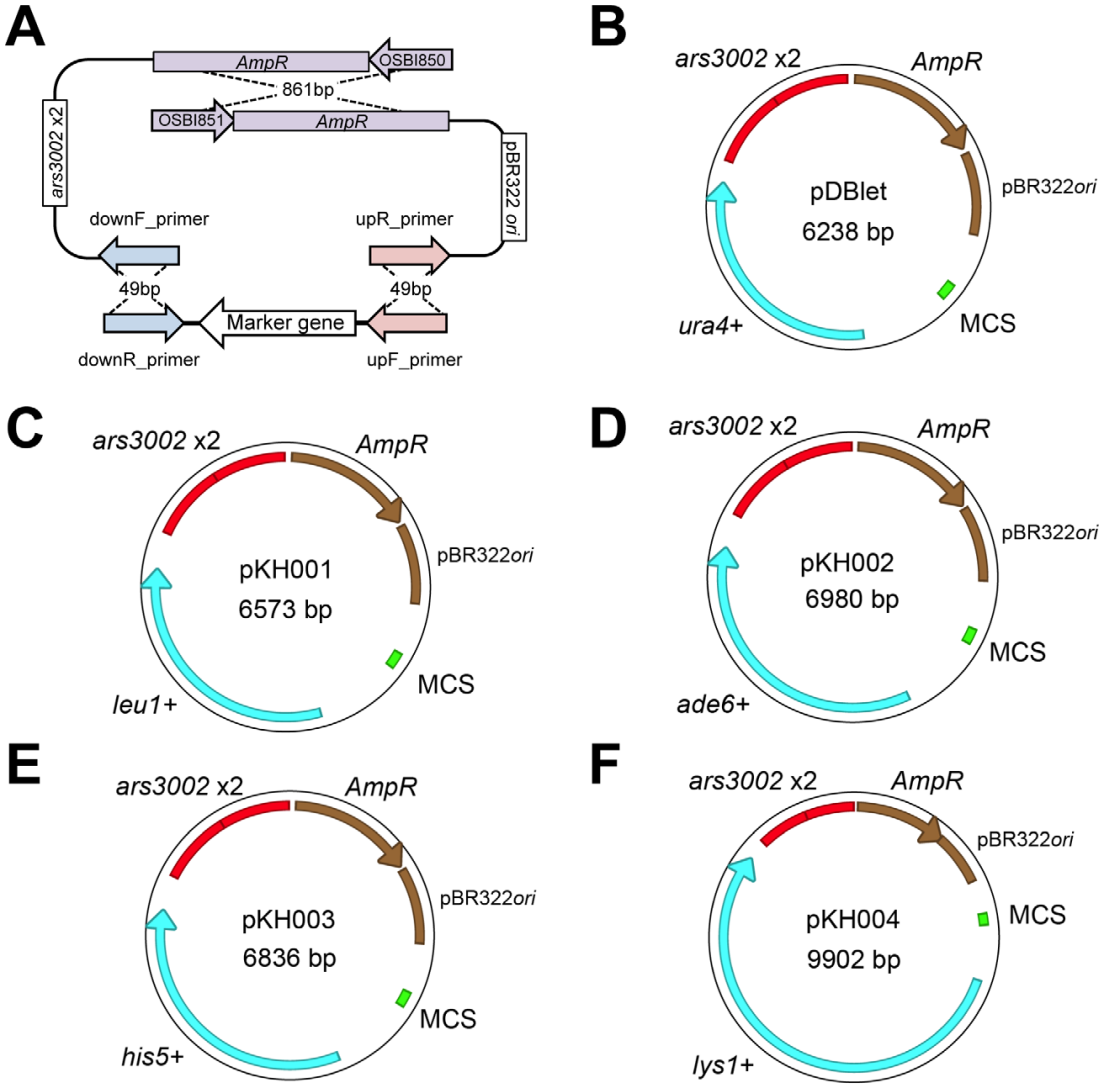
Construction of plasmids with different auxotrophic markers using GRC in *S. pombe.* (**A**) Blueprint of plasmid construction. DNA fragments containing each marker gene and the plasmid backbone were amplified by PCR using the indicated primer sets (the primer names are listed in the Supplementary Information) with 972 h genomic DNA and pDblet as templates, respectively. Homologous regions between the fragments are shown in the same colors. For each marker gene, FY7054 were transformed with 3 PCR fragments (i.e. a marker fragment and 2 plasmid fragments) and the transformants were selected on EMM plates without the amino acid associated with the auxotrophic marker. Plasmids were recovered from the transformants and their structure was checked. (**B**) A map of the original pDblet. (**C–F**) Maps of plasmids constructed in this study.

We successfully constructed plasmids with different auxotrophic markers by replacing the *ura4+* gene on pDblet with *leu1+*, *ade6+*, *his5+*, and *lys1+*, respectively (Figure 5) by applying GRC in *S. pombe*. These plasmids were obtained from NBRP-yeast (http://yeast.lab.nig.ac.jp/). Further details on the plasmids can be found in Text S1, S2, S3, S4.

## Materials and Methods

### *S. pombe* Strains Used in this Study

Sp286h+ (*h+ ade6 ura4-D18 leu1-32*), a haploid progeny of Sp286 (Bioneer), was used as the host strain for the GRC experiments that did not involve construction of pKH plasmids (Figure 5). To construct the pKH plasmids, FY7054 (*h+N ade6-M210 his5-303 leu1-32 lys1-131 ura4-D18*) obtained from NBRP-yeast was used as the host strain. The 972h (*h-*) genome was used as a template for the auxotrophic marker genes during pKH plasmid construction. FY4102 (*h- ura4-D18 leu1-32 ade6-210 lig4*::*Kan^R^*) was used as the host strain for GRC without non-homologous end joining [10].

### Plasmids Used in this Study

pDblet [8] was used as a plasmid vector in the GRC experiment. pRS315 [11] was used as the PCR template for the *S. cerevisiae LEU2* gene. pHM004_nmt1-1, a plasmid containing *nmt1* promoter (from our laboratory stock), was used as the PCR template for the *nmt1* promoter. pKT128 [12] was used as the PCR template for *EGFP*.

### Growth Media and Conditions

*S. pombe* cells were cultured in a manner similar to [13] at 30°C. EMM medium and plates were prepared using EMM broth, powder (MP Biomedicals).

### DNA Methods

High-efficiency transformation of yeast, yeast DNA isolations (Yeast DNA miniprep), and DNA miniprep from *E. coli* were performed in a manner similar to [14] by replacing the culture medium with one more appropriate for *S. pombe*. *E. coli* transformation was performed using a XL1-Blue Electroporation Competent Cell (Agilent Technologies). DNA sequencing was performed using an ABI 3730xl Analyzer.

### Fluorescence Imaging

Images of yeast culture plates with GFP fluorescence were obtained using a LAS4000 lumino-image analyzer (Fujifilm) and were processed using Photoshop CS2 software (Adobe).

### Preparation of DNA Fragments for GRC in *S. pombe*

Preparation of the plasmid vector with restriction enzyme digestion: About 10-µg pDblet prepared with DNA miniprep from *E. coli* was digested with the mentioned restriction enzymes in a 100-µL solution. A total of 2-µL (∼0.2 µg) digested plasmid solution was used directly for yeast transformation. Preparation of DNA fragments by PCR: DNA fragments were amplified using KOD polymerase (Toyobo) according to the manufacturer's protocol in 50-µL solution using the primers listed in the Tables S1 along with the template DNA described previously. A total of 45 µL of the amplified DNA solution (containing about 10-µg DNA) was used directly for yeast transformation. The molar ratio of the digested vector and PCR products was about 1:200∼300.

## Supporting Information

Tables S1 This is a PDF file containing lists of primers used in this study.
(0.07 MB PDF)

Text S1 This is a text file containing the nucleotide sequence of pKH001.
(0.01 MB TXT)

Text S2 This is a text file containing the nucleotide sequence of pKH002.
(0.01 MB TXT)

Text S3 This is a text file containing the nucleotide sequence of pKH003.
(0.01 MB TXT)

Text S4 This is a text file containing the nucleotide sequence of pKH004.
(0.01 MB TXT)
