# Supplementary material for: Plasmid Construction Using Recombination Activity in the Fission Yeast Schizosaccharomyces pombe
Source: PLoS One. 2010 Mar 11;5(3):e9652. doi: 10.1371/journal.pone.0009652 (PMC2836380; doi:10.1371/journal.pone.0009652)
Supplement: Tables S1 — This is a PDF file containing lists of primers used in this study. (0.07 MB PDF) [file pone.0009652.s001.pdf]

Primers to construct pDblet + *LEU2*

Primer Name	Sequence*	Description
OHM001	agctatgaccatgattacgccaagcTTCCTCAACATAACGAG	up primer to amplify *LEU2* with 25bp homologous sequence
OHM002	atttcacacaggaaacagctatgaccatgattacgccaagcTTCCTCAACATAACG	up primer to amplify *LEU2* with 41bp homologous sequence
OHM003	gactcactatagggcgaattgggtaCCAATATATAAATTAGG	down primer to amplify *LEU2* with 27bp homologous sequence
OHM004	gcgcgcgtaatacgactcactatagggcgaattgggtaCCAATATATAAATTAGG	down primer to amplify *LEU2* with 40pb homologous sequence
OHM005	tagtggatcccccgggctgcaggaattcgatatcaagcttCCTCAACATAACGAG	up primer to amplify *LEU2* with 40bp homologus sequence adjucent to vector's *Hin*dIII site

* Lower case: vector sequence, Upper case: insert sequence

Primers to construct pDblet + P*nmt1-EGFP*

Primer Name	Sequence*	Description
OHM014	ttaaaccagcaccgtcacccATGATTTAACAAAGCGACTA	down primer for P*nmt-EGFP* fusion
OHM015	TAGTCGCTTTGTTAAATCATgggtgacggtgctggtttaa	up primer for *EGFP*-P*nmt1* fusion
OHM016	ctcactatagggcgaattgggtaccTGCCGGTAGAGGTGTGGTCA	down primer to amplify *EGFP*
OSBI867	TTGTGTGGAATTGTGAGCGG	up primer to amplify *Pnmt1*
OHM025	TGGGGAGAGAAAACAGGGCA	primer to sequence junction region of P*nmt1* and *EGFP*

* Upper case: *EGFP* sequence, lower case: P*nmt1* sequence in OHM014 and OHM015. Lower case: vector sequence, Upper case: insert sequence in OHM016.

Primers to construct pDblet with different auxotrophic markers

Primer Name	Sequence*	Description
OSBI850	TTACCAATGCTTAATCAGTG	AmpR from Stop
OSBI851	ATGAGTATTCAACATTTCCG	AmpR from Start
OHM133	GCATCTGTGCGGTATTTCACACCGatgtatttttttaagtattacactta	upF primer to construct pKH001 (pDblet-leu1)
OHM134	taagtgtaatacttaaaaaatacatCGGTGTGAAATACCGCACAGATGC	upR primer to construct pKH001 (pDblet-leu1)
OHM135	aattatttctttttaaacgaagtaAAAGCCAATGAAAGATGTATGTAGA	downF primer to construct pKH001 (pDblet-leu1)
OHM136	TCTACATACATCTTTCATTGGCTTTtacttcgtttaaaaagaaataatt	downR primer to construct pKH001 (pDblet-leu1)
OHM137	GCATCTGTGCGGTATTTCACACCGtagtgatacgcacattgaaacatgg	upF primer to construct pKH002 (pDblet-ade6)
OHM138	ccatgtttcaatgtgcgtatcactaCGGTGTGAAATACCGCACAGATGC	upR primer to construct pKH002 (pDblet-ade6)
OHM139	aacgcatattaatgcaaaaaatgcAAAGCCAATGAAAGATGTATGTAGA	downF primer to construct pKH002 (pDblet-ade6)
OHM140	TCTACATACATCTTTCATTGGCTTTgcatttttttgcattaatatgcgtt	downR primer to construct pKH002 (pDblet-ade6)
OHM141	GCATCTGTGCGGTATTTCACACCGcagggaatgacttctgtgaaaaata	upF primer to construct pKH003 (pDblet-his5)
OHM142	tatttttcacagaagtcattccctgCGGTGTGAAATACCGCACAGATGC	upR primer to construct pKH003 (pDblet-his5)
OHM143	gatgcacaaacgtccgattccataAAAGCCAATGAAAGATGTATGTAGA	downF primer to construct pKH003 (pDblet-his5)
OHM144	TCTACATACATCTTTCATTGGCTTTtatggaatcggacgtttgtgcatc	downR primer to construct pKH003 (pDblet-his5)
OHM145	GCATCTGTGCGGTATTTCACACCGtgtgaaaaatgtggttctgattcag	upF primer to construct pKH004 (pDblet-lys1)
OHM146	ctgaatcagaaccacatttttcacaCGGTGTGAAATACCGCACAGATGC	upR primer to construct pKH004 (pDblet-lys1)
OHM147	gaataggaggaggcctacacgactAAAGCCAATGAAAGATGTATGTAGA	downF primer to construct pKH004 (pDblet-lys1)
OHM148	TCTACATACATCTTTCATTGGCTTTagtcgtgtaggcctcctcctattc	downR primer to constructp KH004 (pDblet-lys1)

* Lower case: Marker sequence, Upper case: vector sequence
